# Conventional Versus Digital Radiographs in Detecting Artificial Voids in Root Canal Filling Material

**Published:** 2011-08-15

**Authors:** Mohammad Mahdi Aghdasi, Mohammad Asnaashari, Alireza Aliari, Farahnaz Fahimipour, Sepideh Soheilifar

**Affiliations:** 1. Department of Radiology, Dental School, Shahid Beheshti University of Medical Sciences, Tehran, Iran.; 2. Department of Endodontics, Dental School, Shahid Beheshti University of Medical Science, Tehran, Iran.; 3. Dental Student, Dental Research center, Shahid Beheshti University of Medical Sciences, Tehran, Iran.

**Keywords:** Dental, Digital, Obturation, Radiography, X-Ray Film

## Abstract

**INTRODUCTION:**

Inappropriate condensation of gutta-percha or improper use of sealer can lead to voids in root canal filling material and consequent failure of the treatment. Timely detection of voids within root canal filling may prevent complications. In this study, we compared the accuracy of digital and conventional radiograph for detecting voids within root canal fillings.

**MATERIALS AND METHODS:**

The root canals of 50 extracted maxillary permanent incisors were prepared and filled with gutta-percha and sealer. The teeth were then randomly divided into two groups of 25 incisors. The teeth were imaged using the paralleling technique with E-speed film and digital/digital zoomed system. The accuracy of radiographic techniques was evaluated for detecting voids by three independent observers. Presence/absence of voids was recorded and compared with the baseline data. The sensitivity, specificity, accuracy and positive and negative predictive values was recorded.

**RESULTS:**

The sensitivity, specificity and accuracy of conventional radiography and digital radiography were 48%, 52%, 50%, and 82.7%, 80% and 81.3%, respectively. The positive and negative predictive value of conventional radiography was 50%. Digital images showed the positive predictive value of 80.3% and negative predictive value of 83.5%. The values of positive and negative predictive were reported as 81.6% and 81.1% in digital zoomed images.

**CONCLUSION:**

Digital and digital zoomed images performed better than conventional radiographs in detecting voids, but there were no differences between the performances of both digital images.

## INTRODUCTION

A crucial goal of successful root canal therapy is the precise obturation of the prepared root canal system; however during root canal therapy some inevitable complications may occur. Presence of voids in both apical and coronal parts of root filling is one predicament which might provide pathways for leakage leading to post-treatment failures [1][2].

Clinical radiography is minimally invasive and the only clinical way to evaluate the quality of the treatment; however this technique encompasses some problems [3]. Along with the advancement in digital systems, digital radiography has been introduced as a substitute for the conventional technique. The efficiency of digital imaging in comparison with film-based radiography has been reported in many studies [4][5][6]. Kositbowamchai et al. compared the diagnostic potential of direct digital images with conventional radiographs in detecting simulated root canal voids. They concluded that the diagnostic performance of digital and conventional images in detecting voids of root canal fillings were not significantly different [7]. Huybrechts et al. study compared intraoral analogue, intraoral digital and cone beam computed tomography (CBCT) images in detecting voids present in root canal filling. They concluded that in the case of digital intra oral technique performed better in detecting small voids compared with intraoral analogue and CBCT images [8]. However, voids larger than 300nm were discerned with all imaging techniques. Some have doubts about efficiency of digital radiographs in void detection, and in obturated canals. More studies are required to compare the accuracy of digital images with conventional images.

The aim of the present study was to compare the diagnostic value of conventional radiographs with digital images and to compare digital images with digital zoomed images in detecting voids in root filling material.

## MATERIALS AND METHODS

### Sample preparation

Fifty extracted maxillary permanent incisors were used in this study. They were stored in normal saline until required. The teeth were decoronated at the cemento-enamel junction with a sharp bur. Each root was marked with a radiopaque marker 6mm from the apex with an aluminum foil in order to align the sites of void formation. The root canal lengths were determined by passing a K-file size 10 (Mani Inc., Tochigi-Ken, Japan) inside the canal just through the apical opening. The step back technique was used to prepare root canals with K-files (Mani Inc., Tochigi-Ken, Japan). Canals were instrumented with size 45 as the master apical file (MAF) and up to size 80. Canals were irrigated with normal saline using 25-gauge needle. After drying with paper points, all root canals were filled with the single-cone technique using gutta-percha cones and ADSeal (Mochoong-Dong, Cheongju City, Korea) as the root canal epoxy resin sealer. The gutta-percha was cut with a hot instrument 1mm below the orifices of canals. The filled canals were divided into two groups, 25 filled root canals with simulated voids and 25 without voids. In order to simulate a void, a 0.2mm in diameter blunt needle was heated and inserted into the cone. The locality of the void was adjusted randomly at 5mm intervals starting at the apex.

### Imaging

The root canals were imaged in the bucco-lingual direction using XCP for paralleling technique (XCP; Dentsply, Elgin). The receptor and tooth position were the same in both digital and conventional radiography.

A plaster block was designed to receive the film/receptor, radiographic tube and tooth block helped to keep the receptor and tooth position in a fixed status. All radiographs were exposed with the X-ray unit operating at 65 KVP and 10mA with a 25cm target film distance (Conventional system: Gendex 765DC, Plaines, IL USA, Digital system: RVG, Trophy, Kodak Company, Paris, France). The digital images were recorded on RVG-CCD receptor (Vme, Eastman Kodak Company, Paris, France). Conventional film images were obtained on size number 2 E-speed periapical films (Agfa, Heraeus Kulzer GmbH; Hanau, Germany) and were processed automatically (Gendex; Clarimat300, London, England).

### Void detection

All images were evaluated by three independent observers (one endodontist and two oral radiologists). Observers with a kappa value of ≥0.80 for detecting voids were selected for the study. For each image, the observer was asked to record the presence or absence of a void. Void detection 5mm above gutta-percha tips were considered to have voids (positive). All observers evaluated the images under the same condition. Conventional films were viewed at distance range between 50-100cm, with a 2× magnification and a viewing box that was collimated to the size of the no. 2 periapical films. Digital images were evaluated using the same procedure. They were displayed on a 19 inch monitor set at a 32 bite resolution. The images on the screen were presented without enlargement for the first group and then with 2× enlargement for the improved digital image group.

### Statistical analysis

The number of correct assessments (voids present/absent) were recorded and compared with baseline data. Diagnostic tests were used to analyze the data, which included sensitivity, specificity, and accuracy, positive and negative predictive value. The differences in sensitivity and specificity between the conventional technique and digital systems were then calculated with significance level set at 0.05.

## RESULTS

The results of conventional radiographs, digital images and improved digital images are shown in Table 1.

**Table 1:** Diagnostic value of conventional radiographs, digital images and improved digital images in detecting root canal filling voids

**Diagnostic index**	**Conventional radiographs**	**Digital images**	**Improved digital images**
**False positive**	36	15	13
**False negative**	39	13	15
**True positive**	36	62	60
**True negative**	39	60	62
**Sensitivity**	48%	82.7%	80%
**Specificity**	52%	80%	83.7%
**Accuracy**	50%	81.3%	81.3%
**Positive predictive value**	50%	80.3%	81.6%
**Negative predictive value**	50%	83.5%	81.1%

Results of measuring concordance amount between conventional and digital radiography, conventional and improved digital radiography, and digital and improved digital radiography are shown in Table 2,Table 3,Table 4 respectively.

**Table 2:** Concordance between conventional radiography and digital radiography^a^

**Conventional radiography**	**With void**	**Without void**	**Total**
**With void**	68	10	78
**Without void**	4	68	72
**Total**	72	78	150

^a^ Kappa= 0.81, P<0.0001

**Table 3:** Concordance between conventional radiography and improved digital radiography^a^

**Conventional radiography**	**With void**	**Without void**	**Total**
**With void**	51	27	78
**Without void**	21	51	72
**Total**	72	78	150

^a^ Kappa= 0.36, P<0.0001

**Table 4:** Concordance between digital radiography and improved digital radiography^a^

**Conventional radiography**	**With void**	**Without void**	**Total**
**With void**	51	21	72
**Without void**	21	57	78
**Total**	72	78	150

^a^ Kappa=0.43, P<0.0001

## DISCUSSION

Voids in root canal fillings can affect the outcome of treatment [1]; in addition, detection of voids is a difficult procedure. Radiographs which are taken at the final stage of the treatment can be used for assessing the quality of root canal filling and this is the only paraclinical way to determine the density of the filling and presence of voids [5]. In the present study, diagnostic accuracy of digital and zoomed digital images in detecting voids was compared with conventional radiographs. Results demonstrated that conventional radiographs had the lowest diagnostic value according to the sensitivity, specificity and accuracy with positive and negative predictive values. There were no significant differences between digital and digital zoomed images (P>0.05); however the sensitivity and specificity values of digital and digital zoomed images were different. The sensitivity value of digital images was more than zoomed digital images while the specificity value the opposite was true. Kositbowrnchai et al. [7] studied the diagnostic accuracy of digital and conventional radiographs in detecting voids in eighty extracted incisor teeth, their results concurred with ours. The area of voids in root canal fillings can indicate the prognosis of treatment. Teeth with voids at apical or middle third of root canal have poorer prognosis than those with voids in coronal third or those without voids [6]. The present study focused on void detection in the apical third of root filling.

The size of voids can also influence the survival of the root canal treatment. However, various radiographic techniques detect different void sizes. The simulated void size in this study was 0.2mm; while the average total length of voids per canal was reported 0.9±0.6mm by Da Saliva et al. [9]. It’s clear that the sensitivity of distinguishing smaller voids is higher. Huybrechts et al. compared detection of different void sizes in root filling using various radiographic techniques and found no differences in detecting large voids [8][10]. In our study, the size of simulated void is smaller than average size of voids.

The higher amount of sensitivity, accuracy and specificity in digital images can be attributed to different diagnostic features in digital and conventional radiographs. In order to improve and facilitate diagnosis via conventional radiographs, a magnifying glass can be used. In addition, in conventional radiography, physio-logic limitations of human eye reduce the diagnostic accuracy. It has been proposed that due to the differences between in vitro and in vivo conditions, the performance of different radiographic methods should be investigated [4].

## CONCLUSION

Digital and zoomed digital images were more accurate in detecting voids compared to conventional radiographs; however there were no significant differences between the perform-ances of digital images.
